# Embedding Equity Across the Autism Diagnostic Pathway: A Diversity, Inclusion, and Equality Audit

**DOI:** 10.1192/bjo.2026.11395

**Published:** 2026-06-30

**Authors:** Grace Howse, Rejaul Islam

**Affiliations:** Skylight Psychiatry, Basingstoke, United Kingdom

## Abstract

**Aims:**

Autism spectrum condition (ASC) remains underdiagnosed in adults, particularly among women and individuals from marginalised and inclusion health groups. Contributing factors include masking, cultural stigma and inequities in service design. Skylight Psychiatry is an adult neurodiversity assessment service delivering NHS-commissioned and self-funded care through a hybrid diagnostic model, combining face-to-face ADOS-2 assessments with virtual clinical assessments and developmental history appointments. Although a range of reasonable adjustments and neuroaffirmative practices are embedded within the service, a formal evaluation of equality, diversity, and inclusion had not previously been undertaken.

This audit evaluates the inclusivity of an adult autism assessment service by analysing patient demographics and systematically reviewing assessment pathways, policies, and accessible information to identify strengths and areas for improvement.

**Methods:**

A mixed-methods service audit was conducted. A voluntary demographic questionnaire was distributed to prospective referrals across two geographical cohorts, with quantitative findings compared against UK population demographics. A structured qualitative review examined policies and procedures, assessment pathways, and neuroaffirmative clinical practice and report writing. This was benchmarked against NHS inclusion health principles, NICE guidelines, and inclusive communication principles.

**Results:**

Quantitative findings demonstrated strong representation of women, gender-diverse individuals, and LGBTQIA+ patients compared with national averages, consistent with emerging literature on adult autism and camouflaging. Demographical representation from some ethnic minority and religious groups was lower than expected, suggesting potential cultural or systemic barriers to access. The qualitative review identified strengths in flexible, patient-centred assessment delivery and consistent neuroaffirmative approaches across appointments. Suggested areas for improvement included suggested evaluation of physical accessibility across clinic sites and consideration of the risks of digital exclusion. Findings suggested that there was some inconsistent use of inclusive language in some clinical documentation, which may be mitigated by the introduction of specific training centred around increasing inclusion and reducing potential diagnostic bias.

**Conclusion:**

This audit demonstrates that a hybrid adult autism diagnostic model can support inclusive and patient-centred care when underpinned by reasonable adjustments and neuroaffirmative practice. However, disparities in access persist for some marginalised groups. It was suggested that the service should prioritise improving equality through the introduction of interventions, such as accessible correspondence, easy-read materials, and translated online content, alongside targeted workforce development. These recommendations are likely to improve access and mitigate adverse outcomes associated with delayed or missed diagnosis.

